# Electrochemical Behavior and Direct Quantitative Determination of Paclitaxel

**DOI:** 10.3389/fchem.2022.834154

**Published:** 2022-04-26

**Authors:** Jiaqi Lang, Wenting Wang, You Zhou, Dongqin Guo, Rujie Shi, Nong Zhou

**Affiliations:** ^1^ The Chongqing Engineering Laboratory for Green Cultivation and Deep Processing of Three Gorges Reservoir Area’s Medicinal Herbs, College of Food and Biology Engineering, Chongqing Three Gorges University, Chongqing, China; ^2^ Wanzhou Institute for Food and Drug Control, Chongqing, China

**Keywords:** paclitaxel, quantitative determination, electrochemical behavior, voltammetry, microemulsion

## Abstract

The electrochemical behavior and direct quantitative determination of paclitaxel, a poorly soluble drug made into microemulsion, were researched by cyclic voltammetry in acetate buffer solutions (pH = 4.0) at a glassy carbon electrode. The results show that the oxidation process is irreversible and controlled by diffusion. Moreover, the effects of anodic peak current (Ipa), anodic peak potential, scan rate, pH, and the electrochemical redox mechanism have been studied. The anodic peak current varied linearly with paclitaxel concentration in the range of 5 × 10^−5^ mol/L to 5 × 10^−4^ mol/L, and the detection limit was 9.15 × 10^−8^ mol/L. The results of RSD (0.90%) and recovery (99.22%–101.69%) were obtained. Additionally, it has been proved that one electron and one proton are involved in the electrochemical redox process. The present research has been successfully used to determine paclitaxel in pure and real samples, which further supported the electrochemical behavior investigation of paclitaxel and direct determination of micro-emulsion.

## 1 Introduction

**FIGURE 1:**
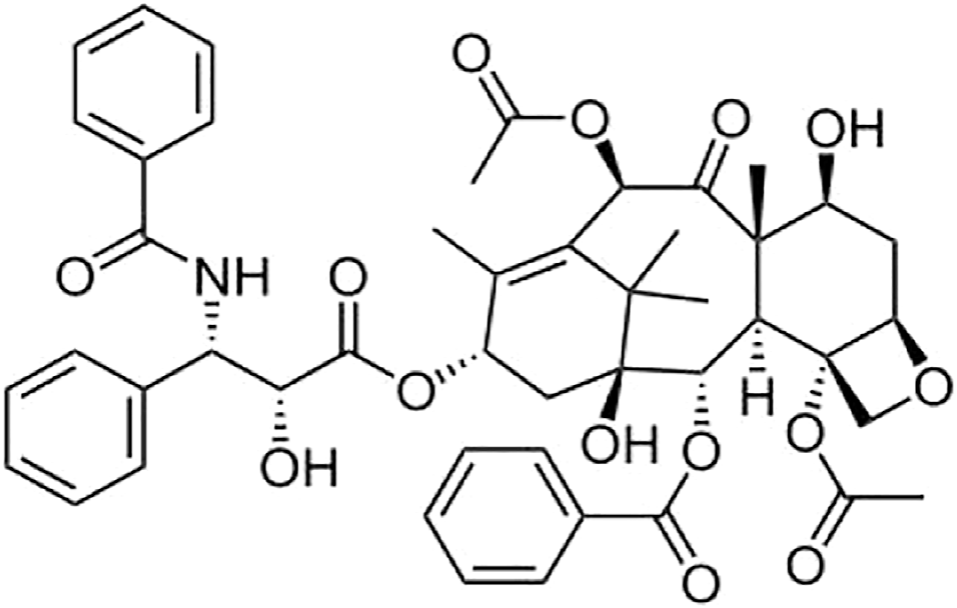
Molecular structure of PAC.

Paclitaxel (PAC; *Taxus brevifolia*, family Taxaceae, molecular structure given in Figure 1) is found and isolated from the bark of Pacific yew by Dr. Wall, Wani et al. for the first time. Paclitaxel (Wani et al., 1971; Ebrahim et al., 2014), a class of tricyclic diterpenoids, is an effective anticancer agent. In the process of cell mitosis, it can prevent the depolymerization of microtubules, thereby inhibiting cell division and proliferation and achieving the effect of killing tumor cells (Zhang et al., 2012). PAC has significant effects on the treatment of metastatic breast cancer, non-small cell lung cancer, ovarian cancer, and head and neck cancer, among others (Spencer and Faulds, 1994; Singla et al., 2002; Yardley, 2013). Nonetheless, PAC is almost insoluble in water or other clinical solvents, limiting its application and development. Correspondingly, in order to increase its solubility, various formulation strategies have been researched and developed, including microemulsion (Fan et al., 2004), nanoparticles (Hawkins et al., 2008), polymer micelles (Ebrahim et al., 2014), cyclodextrin inclusions and compounds (Sharma et al., 1995), and liposomes (Wu et al., 2006).

Furthermore, the microemulsion is a transparent, low-viscosity, isotropic, and thermodynamically stable oil-water mixture formed spontaneously by the water phase, oil phase, and surfactant. Due to the nano-level aggregation of the microemulsion, it has the advantages of good stability, good biocompatibility, and high solubility (Shinoda and Lindman, 1987; Sjöblom et al., 1996; Lawrence and Rees, 2000; Ganguli et al., 2010), it has become increasingly important in application areas such as pharmaceutical research and development, biotechnology, daily chemical industry, and chemical reactions, especially for containing functional additives such as drugs, polymers, and nanoparticles (Magdassi, 1997; Lawrence and Rees, 2001; Acharya and Hartley, 2012).

The commonly used clinical paclitaxel injection (trade name: Taxol) is a kind of microemulsion, which is mainly solubilized with polyoxyethylene castor oil (Cremophor EL) anhydrous ethanol (V:V = 50:50).

At present, there are a large number of methods to quantify PAC, including HPLC, HPLC-MS, and UPLC-MS (Bonde et al., 2019; Christner et al., 2019; Attia et al., 2020), which take a long time and cost a lot. On the contrary, there are very few reports on the quantization of paclitaxel by electrochemical methods (Gowda and Nandibewoor, 2014). Therefore, it is urgent to improve and develop a more rapid and efficient electrochemical analysis method for the quantitative study of paclitaxel.

In general, this study aims to establish a simple, efficient, fast, and low-concentration method for detecting paclitaxel by cyclic voltammetry at the surface of glassy carbon electrode (GCE) and further investigate the electrochemical behavior and redox mechanism of paclitaxel micro-emulsion. Specifically, the experimental parameters such as supporting electrolyte and its concentration, the effects of anodic peak current (Ipa), anodic peak potential, scan rate, and pH were optimized. Meanwhile, the electrochemical mechanism, including the amount of electron transfer reaction, is studied. The obtained results can provide certain theoretical and practical application value for the electrochemical behavior research and rapid content determination of insoluble drugs.

## 2 Materials and Methods

### 2.1 Materials and Reagents

Paclitaxel was purchased from Taihao Pharmaceutical Co., Ltd (Chongqing, China). Paclitaxel injection (Taxol), a clear and colorless solution, was purchased from Bristol-Myers Squibb, United States (30 mg in 5 ml). Other reagents and materials were provided by Chengdu Kelon Chemical Reagent Co., Ltd. (Sichuan, China) and were of analytical reagent (AR) grade. All solutions are prepared in ultrapure water.

### 2.2 Apparatus

CHI610E electrochemical workstation (Shanghai, China) is used for electrochemical measurement. The electrode system consists of three parts, the working electrode is a glassy carbon electrode (3 mm in diameter), the counter electrode is a platinum electrode, and the reference electrode is a saturated calomel electrode. In order to improve the reproducibility and sensitivity of the peak current, the electrode was cleaned five times in ethanol using cyclic voltammetry before each measurement.

### 2.3 Procedure

#### 2.3.1 Sample Preparation

A standard stock solution (1.0 mmol/L) of PAC was prepared in methanol. Accurately measure 1.4 ml of Taxol and dilute to 100 ml with methanol as a sample stock solution (1.0 mmol/L) and store in a cool place. The test solution used for the electrochemical experiment consisted of 2 ml supporting electrolyte and 2 ml stock solution. In addition, the test solution was deoxygenated by purging with nitrogen for 5 min before the measurement.

#### 2.3.2 Voltammetry Parameters

All electrochemical experiments were performed at 25 ± 0.1°C. The parameters of cyclic voltammetry are the initial potential E, which is −1.5 V; the low initial potential E is 0.5 V; the sensitivity is 0.01 mA/V; the standing time is 2 s; and the scan rate is 50 mV/s.

### 2.4 Area of Electrode

The area of the glassy carbon electrode was obtained by cyclic voltammetry using 1 mM K_3_Fe(CN)_6_ as a probe at different scan rates. For a reversible process, the following Randles–Sevcik formula can be used (Goyal and Bishnoi, 2011):

$$I_{pa}=0.4463{\left({\text{F}}^{3}/RT\right)}^{1/2}{\text{An}}^{3/2}{\text{DR}}^{1/2}{\text{C}}_{0}v^{1/2},$$ (1)

where *F* = 96,485 C/moL, *R* = 8.314 J/mol K, *T* is the absolute temperature (298 K), *A* is the surface area of the electrode (cm^2^), *I*_*pa*_ refers to the anodic peak current (Ampere), and *n* is the number of electrons transferred. For 1 mM K_3_Fe(CN)_6_ in 0.1 M KCl electrolyte*, D*_*R*_ = 7.6 × 10^–6^ cm^2^ s^−1^, *n* = 1, *C*_*0*_ is the concentration of K_3_Fe(CN)_6_ (mol/L) and *v* is scan rate (V/s), respectively. The surface area was calculated from the slopes of the plot of the I*pa* vs. *v* ^1/2^, and the area of the glassy carbon electrode was calculated to be 0.0548 cm^2^.

## 3 Results

### 3.1 Selection of Supporting Electrolyte

The electrochemical detection sample peak is obviously affected by the electrolyte. Therefore, the electrolyte was screened for test conditions using 5.0 mol/L acetate buffer (pH 4.0), B-R buffer solution (pH 4.0), 0.1 mol/L NaOH, citric acid-sodium citrate (pH 4.0), 0.5 mol/L KCl, 0.1 mol/L HCl, 0.5 mol/L phosphate buffer solution, and 0.1 mol/L H_2_SO_4._ The test solution used for the electrochemical experiment consisted of 2 ml supporting electrolyte and 2 ml stock solution. It was found that the voltammetry peak obtained with pH 4.0 5 M acetate buffer was the best. This composition of the solution is necessary because acetic acid has a relatively low dielectric constant (ε = 6.15 at 25°C), which causes a slight dissociation of the electrolyte and further reduces the ohmic potential significantly (Michalkiewicz, 2011).

### 3.2 Cyclic Voltammetric Studies of Paclitaxel

**FIGURE 2:**
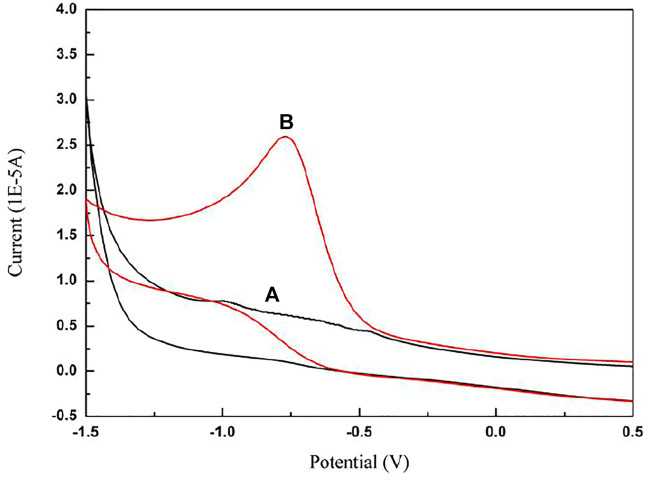
Cyclic voltammograms of **(A)** 5.0 mol/L acetate buffer (pH 4.0) in the absence of paclitaxel, **(B)** 5.0 mol/L acetate buffer (pH 4.0) in the presence of paclitaxel (5 × 10^−4^ mol/L) at GCE; scan rate is 50 mV/s.

In the absence and presence of paclitaxel, the cyclic voltammogram of the electrochemical behavior recorded on the GCE, in which the electrolyte is 5.0 mol/L acetate buffer (pH 4.0) and the scanning speed is 50 mv/s, is shown in Figure 2. The experimental results show that, in the absence of paclitaxel, the electrode reaction cannot occur within the potential range of 0.5 V to −1.5 V. After adding paclitaxel stock solution to the solution, only one obvious irreversible anode peak appeared in the potential range from −0.6 to −0.8 V, indicating that an oxidation reaction occurred and the oxidation product no longer had electrical activity. However, the oxidation process is irreversible because no peaks are observed during the reverse scan.

### 3.3 Effect of pH

**FIGURE 3:**
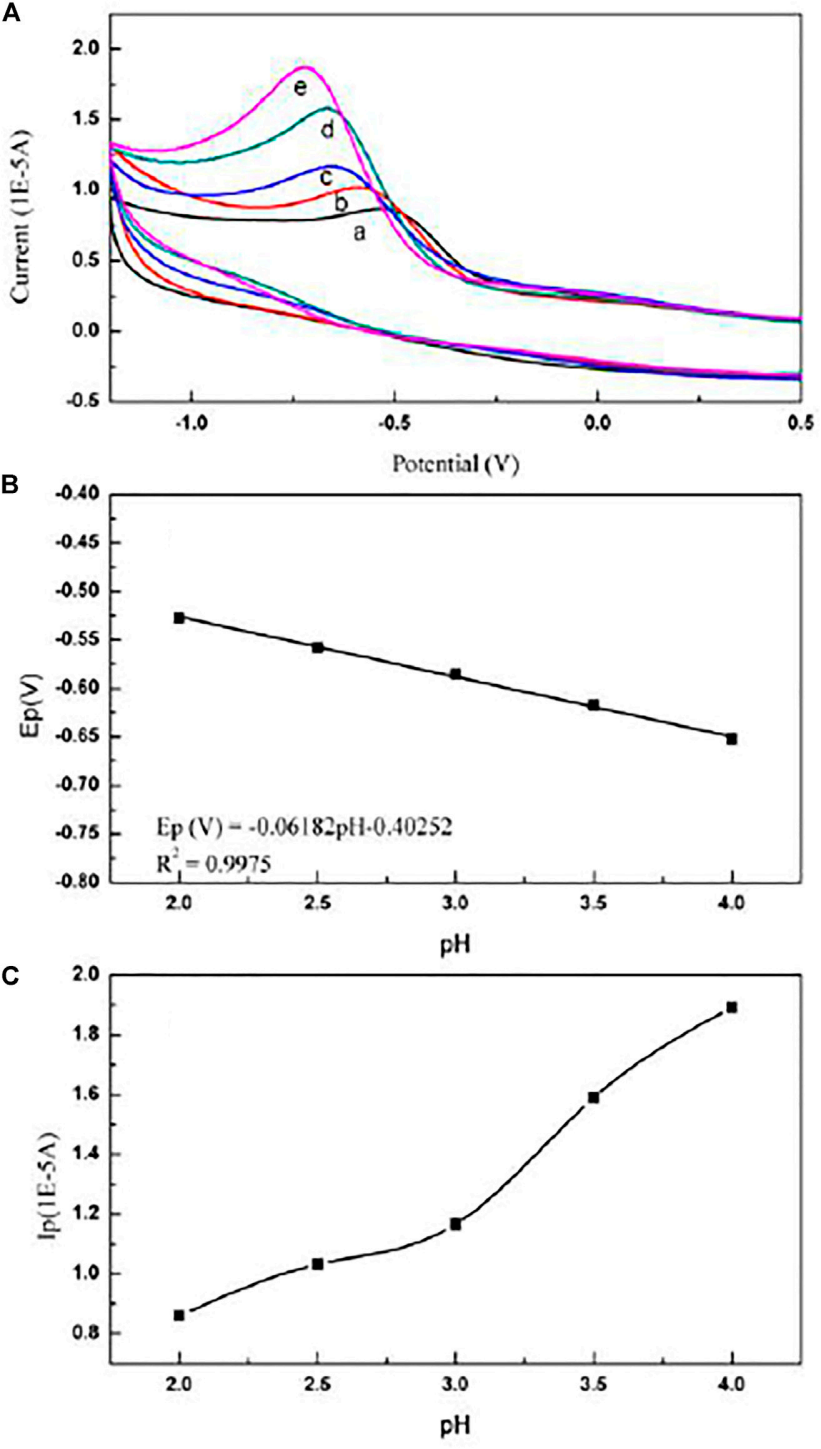
**(A)** Cyclic voltammogram of paclitaxel in electrolytes with different pH: **(a)** pH = 2.0; **(b)** pH = 2.5; **(c)** pH = 3.0; **(d)** pH = 3.5; **(e)** pH = 4.0. **(B)** Dependence of pH on the potential Ep (V) of paclitaxel. Scan rate is 50 mV/s. The concentration of paclitaxel is 1 × 10^−5^ mol/L. **(C)** Dependence of pH on the current Ip (V) of paclitaxel. Scan rate is 50 mV/s. The concentration of paclitaxel is 1 × 10^−5^ mol/L.

While the pH value of the supporting electrolyte is greater than 4.0, the mixed solution of paclitaxel microemulsion and supporting electrolyte is turbid, possibly because the increasing pH destroys the formation of the microemulsion and demulsification occurs. The effect of pH on the cyclic voltammogram of paclitaxel is shown in Figure 3.

Figure 3A and Zhang et al. (2012) showed that, in the pH range from 2.0 to 4.0, as the pH value of the supporting electrolyte increases, the peak potential of the oxidation peak shifts to a negative potential. At the same time, the linear relationship between the peak potential of paclitaxel and the pH of the supporting electrolyte is as follows:

$$\text{Ep}\left(\text{V}\right)=-0.06182\;\text{pH}-0.40252\left({\text{R}}^{2}=0.9975\right).$$ (2)

The slope of this equation is 61.82 mV/pH, which is close to the expected theoretical value of 59 mV/pH (Cisternas et al., 2015), further indicating that the number of protons and electrons involved in the reaction is equal.

On the contrary, it can be seen from Figure 3C that when the pH range is from 2.0 to 4.0, the peak current value of the cyclic voltammogram increases with the increase in pH. Therefore, an electrolyte of pH 4.0 was selected for further testing.

### 3.4 Effect of Scan Rate

**FIGURE 4:**
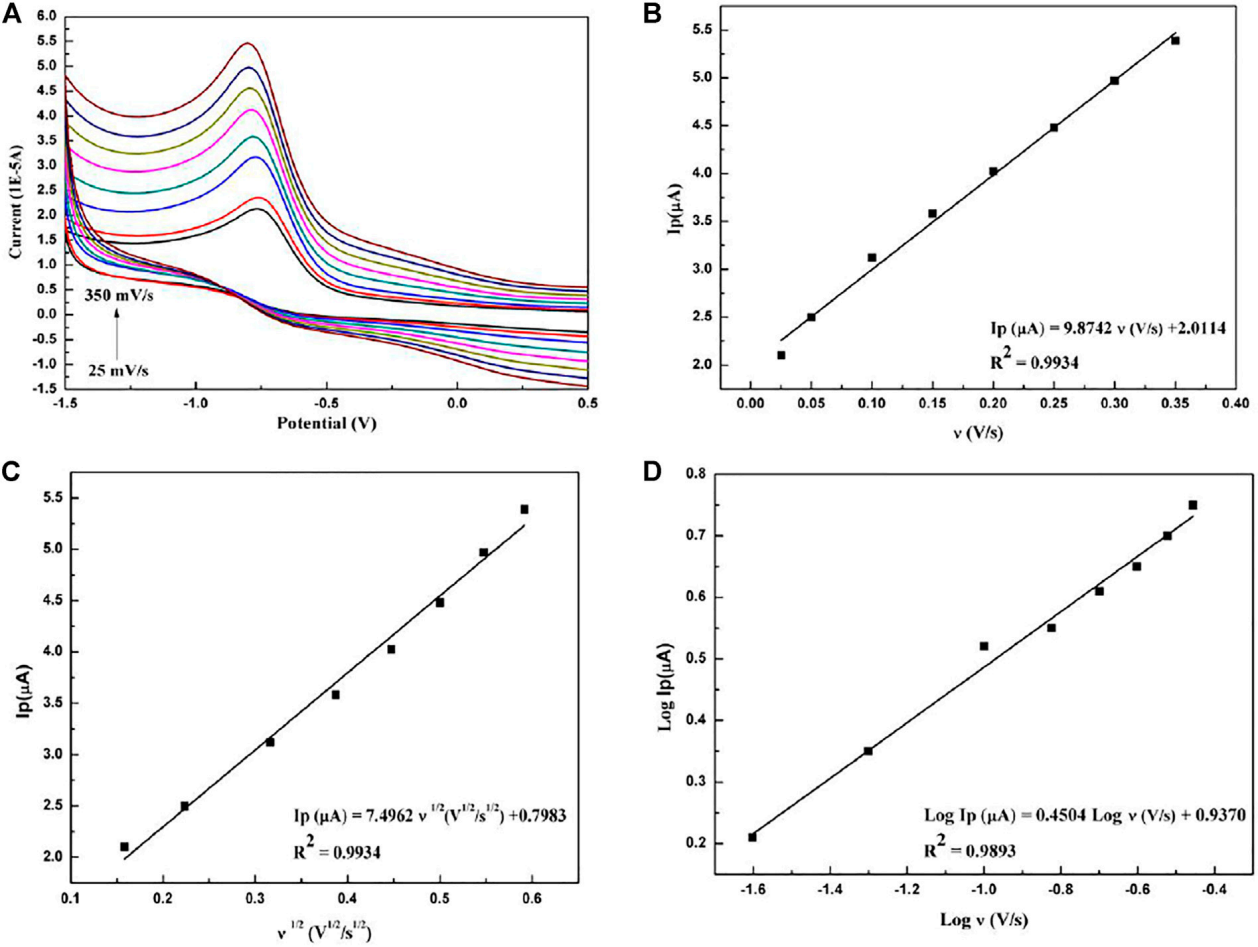
**(A)** Cyclic voltammograms of paclitaxel (1 × 10^−5^ mol/L) with different scan rates: 0.025, 0.05, 0.10, 0.15, 0.20, 0.25, 0.30, and 0.35 V/s. **(B)** Dependence of peak current on scan rate. **(C)** Dependence of peak current on the square root of scan rate. **(D)** The linear relationship between the logarithm of peak current and logarithm of scan rate.

The electrochemical oxidation-reduction reaction mechanism can be obtained from the relationship between the scan rate and the cyclic voltammogram. In the range of the scanning speed of 0.025 V/*s* to 0.35 V/*s*, the relationship between the electrochemical peak and the scanning speed is shown in Figure 4. Figure 4B shows that the scan rate and peak current form a good linear relationship, described as follows:

$$I_{p}\left(\mu \text{A}\right)=9.8742\;v\;\left(\text{V}/\text{s}\right)+2.0114\left(R^{2}=0.9934\right).$$ (3)

It shows that the oxidation reaction of paclitaxel is controlled by adsorption (Wu et al., 2016). Meanwhile, the relationship between the square root of the scanning speed and the peak current, which is shown in Figure 4C, is as follows:

$$I_{p}\left(\mu \text{A}\right)=7.4962\;v^{1/2}\left({\text{V}}^{1/2}/{\text{s}}^{1/2}\right)+0.7983\left(R^{2}=0.9934\right).$$ (4)

The square root of the scanning speed and the peak current form a good linear relationship, indicating that the reaction is also a typical diffusion control process (Prashanth et al., 2011). In addition, Figure 4D shows that the logarithm of the peak current has a good linear relationship with the logarithm of the scan rate and satisfies the following equation:

$$\text{Log}\;I_{p}\left(\mu \text{A}\right)=0.4504\;\text{Log}\;v\left(\text{V}/\text{s}\right)+0.9370\left(R^{2}=0.9893\right).$$ (5)

The correlation slope is 0.45, close to the theoretical value of 0.5, further indicating that the oxidation reaction has a diffusion process (Chrzescijanska et al., 2014).

**FIGURE 5:**
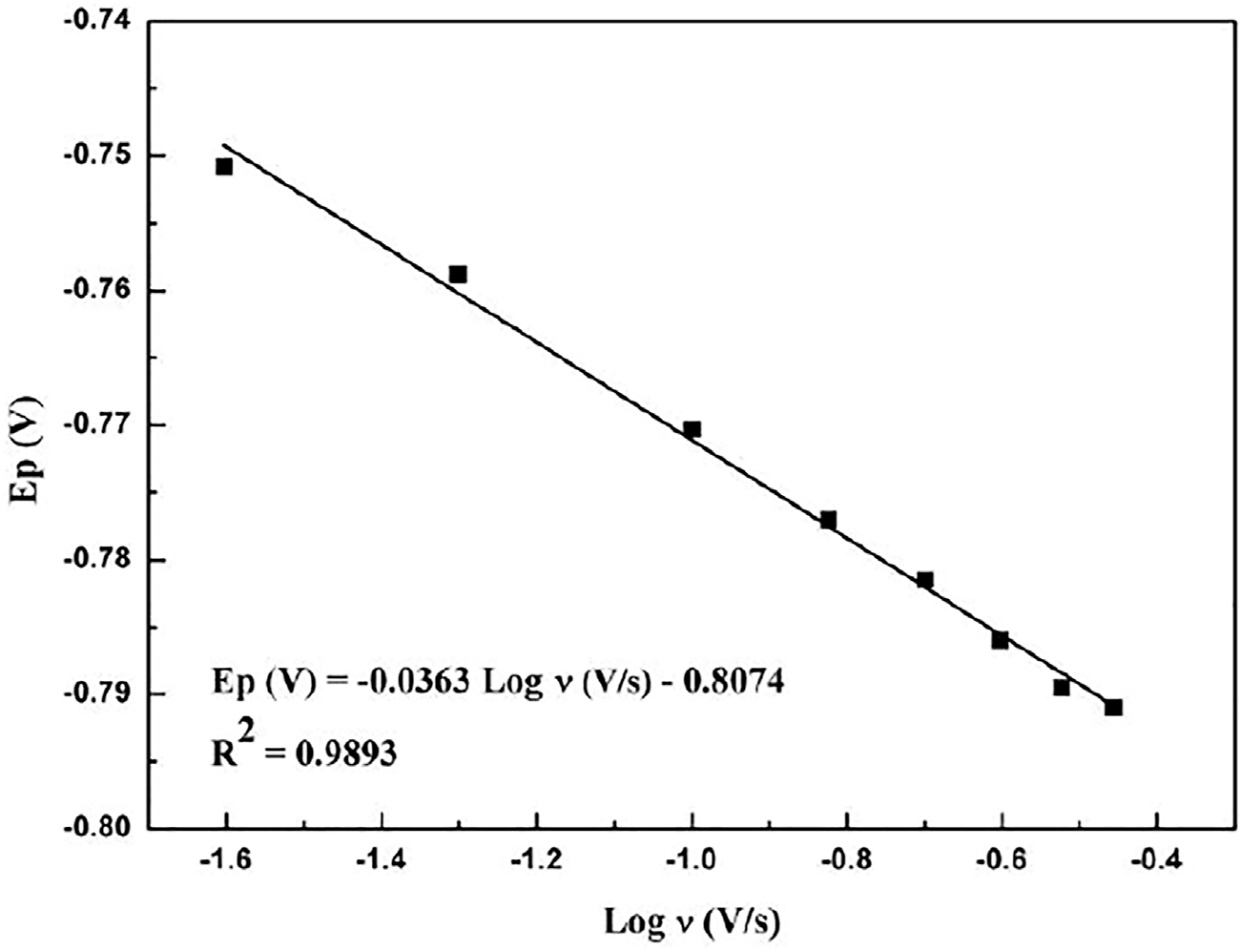
Linear relationship with redox peak potential and logarithm of scan rate.

Figure 5 shows the relationship between the peak potential of paclitaxel and the logarithm of the scan rate. The linear relationship is as follows:

$$E_{p}\left(\text{V}\right)=-0.0363\;\text{Log}\;v\left(\text{V}/\text{s}\right)-0.8074\left(R^{2}=0.9893\right).$$ (6)

It can be seen that, as the scanning speed increases, the peak potential becomes more negative, indicating that the electrochemical reaction is a standard irreversible process (Chrzescijanska et al., 2014; Wu et al., 2016).

For irreversible electrochemical reactions, *Ep* can be obtained by the following formula (Chrzescijanska et al., 2014):

$$E_{p}\left(\text{V}\right)=\left(-2.3RT/\alpha nF\right)\text{Log}\;v\left(\text{V}/\text{s}\right)+\text{constant,}$$ (7)

where *n* is the total number of electrons transferred, *α* is the electron transfer coefficient, *R* = 8.314 J/K mol, *F* = 96,480 C/mol, and *T* = 298 K. From the slope of *E*_*p*_ and log *ν* (−0.0363), the value of *αn* can be calculated as 1.627 and *α* can be obtained by the following formula (Bard and Faulkner, 2004):

$$\alpha =47.7/\left(E_{p}-E_{p/2}\right),$$ (8)

where *E*_*p/2*_ (mV) is the potential where the current is at half the peak value. *α* was calculated to be 0.892, so *n* was approximately calculated to be 2, indicating that one electron was involved in the electrochemical process of paclitaxel. The apparent heterogeneous electron transfer rate constant κ_s_ can be obtained according to the following equation (Laviron, 1979):

$$\kappa_{s}=\alpha nFv_{c}/RT,$$ (9)

where *ν*_*c*_ is the selected speed rate, which is 0.05 V/s, and κ_s_ is final calculated to be 3.17 s^−1.^

### 3.5 Mechanism

**FIGURE 6:**
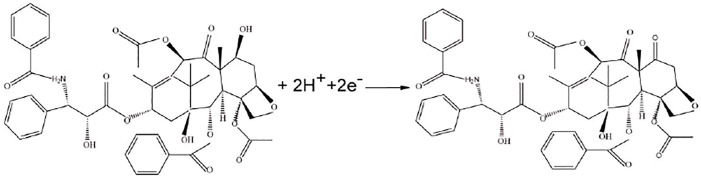
Electrochemical mechanism of paclitaxel.

Previous studies have proved that the electrochemical reaction of paclitaxel involves the transfer of two electrons and two protons, which may be due to the oxidation of the C-7 hydroxyl group of paclitaxel to a carbonyl group. The mechanism is shown in Figure 6. The experimental conclusions are consistent with the existing literature (Prezioso et al., 1995; Jayant and Nandibewoor, 2014).

### 3.6 Determination of Paclitaxel

**FIGURE 7:**
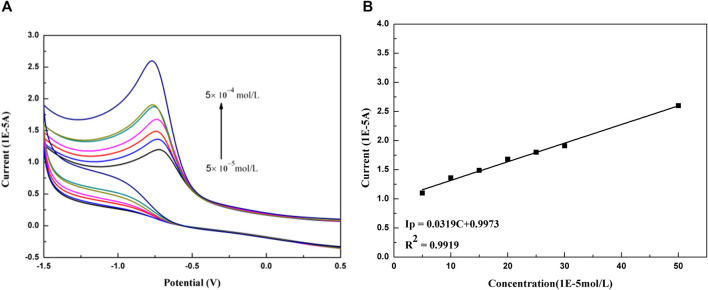
**(A)** Peak current changes with increasing concentration of paclitaxel at GCE (× 10^−5 ^mol/L): 5, 10, 15, 20, 25, 30, and 50. **(B)** Linear relation between current and the concentration of paclitaxel.

The content of paclitaxel microemulsion was determined by cyclic voltammetry. Within the concentration range from 5 × 10^−5^ mol/L to 5 × 10^−4^ mol/L, the change of peak current with paclitaxel concentration is shown in Figure 7. As a result, the linear relationship is good, and the linear equation is *I*_*p*_ (1E-5A) = 0.0319 *C* (1E-5 mol/L) + 0.9973 (*R* ^*2*^ = 0.9919).

The accuracy of the method is evaluated by repeatability and reproducibility. The repeatability is obtained by repeating the experiment five times under the same conditions on the same day; and the reproducibility is obtained by repeating five experiments within 5 days for different people. The experimental results are shown in Table 1. The limit of detection (LOD) and limit of quantifications (LOQ) were calculated by *LOD* = 3 s/*m* and *LOQ* = 10 s/*m* (Blanc et al., 2000)*,* which were to be 9.15 × 10^−8^ mol/L and 3.05 × 10^−7^ mol/L, respectively.

**TABLE 1:** Characteristics of determination of paclitaxel by CV.

Parameter	
Regression equation	Ip (1E-5A) = 0.0319 C (1E-5 mol/L) + 0.9973
Linear range (mol/L)	5 × 10^−5^–5 × 10^−4^
Correlation coefficient (R^2^)	0.9919
LOD (mol/L)	9.15 × 10^−8^
LOQ (mol/L)	3.05 × 10^−7^
Repeatability of peak current (RSD %)	0.32
Repeatability of peak potential (RSD %)	0.17
Reproducibility of peak current (RSD %)	0.64
Reproducibility of peak potential (RSD %)	0.55

### 3.7 Recovery Test

Perform recovery experiments on the established method. The experiment used Taxol (30 mg in 5 ml), and the recovery results calculated by adding a known amount are shown in Table 2. It can be seen that the method has a good recovery (99.22%–101.69%) and acceptable precision (RSD = 0.90%), indicating that the method is feasible.

**TABLE 2:** Recovery of paclitaxel.

Added (mol/L)	Found (mol/L)	Recovery (%)^a^
6.0 × 10^−5^	5.9893 × 10^−5^	99.82
8.0 × 10^−5^	7.9933 × 10^−5^	99.92
1.0 × 10^−4^	1.0169 × 10^−4^	101.69
2.5 × 10^−4^	2.4819 × 10^−4^	99.28
5.0 × 10^−4^	5.0028 × 10^−4^	100.06
8.0 × 10^−4^	7.9376 × 10^−4^	99.22
RSD (%)	0.90	

a Recovery (%) = (mean/added) ×100%.

### 3.8 Effect of Excipients

This method is necessary to study whether excipients interfere with the test results. On the one hand, 5% glucose and 0.9% NaCl are commonly used as excipients, with little interference on the peak current and peak potential of the cyclic voltammogram. On the other hand, the polyoxyethylene castor oil and ethanol used in Taxol’s prescription also have less interference. Therefore, it shows that when the excipients are water, 5% glucose, and 0.9% NaCl, this method can be used in this prescription system. The effect of excipients on potential is shown in Table 3.

**TABLE 3:** Effect of excipients on potential in 1.0 × 10^−4^ M PAC.

Excipient	Potential	Signal change (%)
Water	−0.753	0
5% Glucose	−0.762	1.2
0.9% NaCl	−0.759	0.8

### 3.9 Application

Three batches of Taxol samples were reconstituted with water, and 2 ml of sample solution and 2 ml 5.0 mol/L acetate buffer (pH 4.0) were taken to determine the content by this method. As shown in Table 4, the results indicated that this method could be used to determine paclitaxel in real samples.

**TABLE 4:** Application of CV method in determination of Taxol.

Sample number	True content (mg)	Determination (mg)^a^	Recovery (%)	RSD (%)
1	30.00	29.9716	99.91	0.65
2	30.00	29.9958	99.99	0.12
3	30.00	30.0144	100.05	0.44

a Average of five determinations.

## 4 Discussion

In this study, an electrochemical method was established to determine the content of paclitaxel, and the electrochemical behavior of paclitaxel on the carbon electrode in 5.0 mol/L acetate buffer (pH 4.0) was studied by cyclic voltammetry. It indicated that the reaction is an irreversible process and is controlled by diffusion and adsorption at the same time. In addition, the electrochemical reaction process of paclitaxel involves the transfer of two electrons and two protons and is oxidized. At the same time, the established method was validated with good linearity, recovery, accuracy, and RSD. This method is convenient, fast, and sensitive. The experimental results of this study provide a constructive method for determining the content of the hydrophobic drug paclitaxel and provide a basis for the electrochemical method to study hydrophobic drugs.

## Data Availability

The original contributions presented in the study are included in the article/Supplementary Material, further inquiries can be directed to the corresponding authors.
